# Exploring the intersections of structural inequities and health disparities: the challenge and opportunity of recognizing racism as a public health crisis

**DOI:** 10.1186/s12889-023-16359-3

**Published:** 2023-07-25

**Authors:** Helen-Maria Lekas, Daniel López-Cevallos, Ash Routen

**Affiliations:** 1 Nathan Kline Institute for Psychiatric Research and NYU School of Medicine, Orangeburg, NY, USA; 2 Department of Health Promotion and Policy, School of Public Health and Health Sciences, University of Massachusetts Amherst, 305 Arnold House 715 North Pleasant Street, Amherst, MA 01003 USA; 3 Diabetes Research Centre, University of Leicester, Leicester, UK

## Abstract

Although increasingly being recognized as a driver of poor health and health inequities, there is limited research on the pervasive effects of racism on population health. In this editorial, we set the context and invite contributions for a *BMC Public Health* Collection of articles titled, “Racism as Public Health Crisis.”

The Covid-19 pandemic exposed long standing health and health care inequalities for many underserved populations around the world, such as people living in resource poor areas, ethnically/racially minoritized communities, and people with disabilities [1, 2]. While there has been much attention on health and wellbeing disparities, only recently the academic, health care, media, and political sectors have begun to focus on how structural racism and other intersecting forms of discrimination generate health inequities, including the greater burden of the pandemic for ethnically/racially minoritized communities [3]. Racism and discrimination are pervasive across societies and institutions, and health systems are no different. To promote health and wellbeing equity, it is imperative that we address racism and other forms of structural injustice in laws and policies, research, and practice.

Most of the research on racial/ethnic injustice to date has focused on examining individual and interpersonal expressions of racism and discrimination, and far less attention has been paid to macro “higher-level” determinants operating at the institutional, community, societal, law and policy levels. Furthermore, public health surveillance and monitoring systems continue to focus on individual rather than structural determinants [4]. Recent examples of more “upstream” analyses include work connecting current socioeconomic and health inequities with home lending policies (i.e., redlining) that historically have disproportionately impacted communities of color in urban centers across the United States [5], and environmental racism and social exclusion depriving marginalized communities from safe water and sanitation in high income countries, ranging from the Black and poor residents of Michigan, USA, to the Roma communities in Europe [6].

Recent special issues on structural racism indicate a novel interest in recognizing racism and other forms of structural exclusion as a public health crisis, one that demands a paradigmatic shift in theory, research, and practice. At the core of this shift is the adoption of a socio-ecological, multilevel, decolonial and intersectional approach that places individual and interpersonal experiences of discrimination in the context of community, societal, and global forces (both historical and contemporary) that have systematically excluded entire populations minoritized due to race, gender, class, and geographic location among other intersecting statuses.

Moving forward, to ensure health equity policies, programs, and interventions are based on reliable, effective research we must revisit the limitations of the field of public health to date, including the type of data, guiding theories, and approaches we have used. First, more racially and ethnically representative datasets are needed; whether they are constructed through macro surveillance systems or smaller scale research studies [7]. Underrepresentation of ethnically/racially minoritized populations in public health and clinical research is a continuous problem that limits the application of findings and the ability to conduct comparisons across studies and countries. For example, a review of cardiovascular outcome trials among populations with type 2 diabetes across Europe, the USA, and the UK reported that of eight trials that included race/ethnicity data, South Asians populations (who have a higher diabetes risk compared to White groups) were underrepresented compared to the UK type 2 diabetes population and expected proportions globally [8].

A related limitation of the extant literature stems from the diverse definitions of race and ethnicity across studies even within the same country context. More than two decades ago, Thompson Fullilove suggested reconsidering the utility of classifying individuals by race for health research and, instead, focusing on measuring racism - an idea that deserves further exploration [9]. Although this phenomenon of underrepresentation has been attributed to potential participants’ language barriers and cultural differences, we urge researchers to examine their own explicit and implicit biases that drive the systematic exclusion of communities of color across the research continuum, from design to dissemination of results and application of findings to real world contexts [10].

Moreover, as we are trying to accurately measure and holistically assess structural racism, we suggest adopting mixed-methods approaches and especially, community-centered research methods. Centering community in the research enterprise can elicit the insights and experiences of the communities impacted by racism that are especially valuable in developing reliable measures, and appropriate interventions, programs, and policies to tackle racism. Community-centered approaches may range from increasing participation in clinical trials [11], to methods such as institutional ethnography, which can shed light into how health and other systems discriminate against minoritized patients through policies, regulations, and routine ways of providing care [12]. Lastly, the field may benefit from stronger grounding in social science theories that account for macro and micro processes, including the social production of ill health [13].

Lastly, as part of the paradigm shift in how we study and address racism and health disparities, we suggest there is a need to rethink what populations and phenomena are significant for public health research and interventions. The field tends to associate high prevalence of diseases and occurrences of health-related phenomena with high public health significance. This way of thinking entails the risk of overlooking rare phenomena and diseases that, although affecting a small proportion of patients, are highly salient in understanding how racism and other forms of discrimination produce inequities. Merton’s concept of “strategic research events” (SREs) can be useful in identifying new questions and phenomena related to racism and health. SREs are events, phenomena, and populations that when examined reveal with exceptional clarity the fundamental processes that are at work and effectively promote our understanding of ongoing recalcitrant challenges (such as racism in health care) [14]. One such example is the phenomenon of patient-directed discharges (that is, a patient leaving the hospital before their providers’ recommendation) that remains under-explored. Although these discharges represent only 1–2% of all annual hospital discharges in the U.S., they systematically occur among the most vulnerable inpatients, including Black, young, men who are uninsured, and/or patients with behavioral health issues. As we embark on studying and addressing structural racism and health inequities, we urge our colleagues to identify such events which can shed light on less obvious mechanisms that produce and reproduce health inequities.

In this collection in *BMC Public Health*, we are inviting submissions from around the world relating to ‘Racism as a Public Health Crisis.’ More details can be found here: https://www.biomedcentral.com/collections/RPHC. We are interested in contributions that address experiences of racism, the effects of racism on health, racism as a barrier to health equity, public health policies addressing systemic racism, and the impact of social inequalities on racial disparities. Relevant issues may include social segregation; inequalities in education, employment, and income; carceral systems and policing; health care systems; environmental factors and immigration.

We welcome all methodological approaches and critiques, and particularly encourage authors from the Global South to submit their work. We are keen for authors to consider and acknowledge both the historical context and contemporary implications of their countries’ systems and policies that continue to impact racialized minorities. Lastly, submissions that move beyond diagnosing the issue towards proposing or evaluating policy, environmental or systemic change are needed.

We hope that this Collection will provide a platform for shared learning on the drivers and consequences of racism on public health, highlighting the urgency of the issue, and providing solutions towards addressing its pervasive effects among racialized communities around the world.

## Data Availability

Not applicable.
